# Mitogen-activated protein kinase signaling in plant pathogenic fungi

**DOI:** 10.1371/journal.ppat.1006875

**Published:** 2018-03-15

**Authors:** Cong Jiang, Xue Zhang, Huiquan Liu, Jin-Rong Xu

**Affiliations:** 1 NWAFU-Purdue Joint Research Center, College of Plant Protection, State Key Laboratory of Crop Stress Biology for Arid Areas, Northwest A&F University, Shaanxi, P. R. China; 2 Dept. of Botany and Plant Pathology, Purdue University, West Lafayette, Indiana, United States of America; McGill University, CANADA

Like in other eukaryotic organisms, mitogen-activated protein (MAP) kinase cascades play important roles in response to host and environmental signals in fungal pathogens. In general, mitogen-activated protein kinase (MAPK) is activated by phosphorylation at the well-conserved threonine-x-tyrosine (TXY) motif by mitogen-activated protein kinase (MEK), which is in turn activated by mitogen-activated protein kinase (MEKK). The budding yeast *Saccharomyces cerevisiae* has five MAPK pathways that regulate mating, invasive growth, cell wall integrity, osmoregulation, and ascospore formation. Except for ascosporogenesis-specific MAPK sporulation-specific mitogen-activated protein kinase (Smk1), other yeast MAPKs are conserved in plant-pathogenic ascomycetes to regulate different infection and developmental processes, which is the focus of this review. In phytopathogenic basidiomycetes, MAPKs have only been well characterized in *Ustilago maydis*.

## The Fus3/Kss1 orthologs regulate appressorium formation and other infection processes

**Fig 1:**
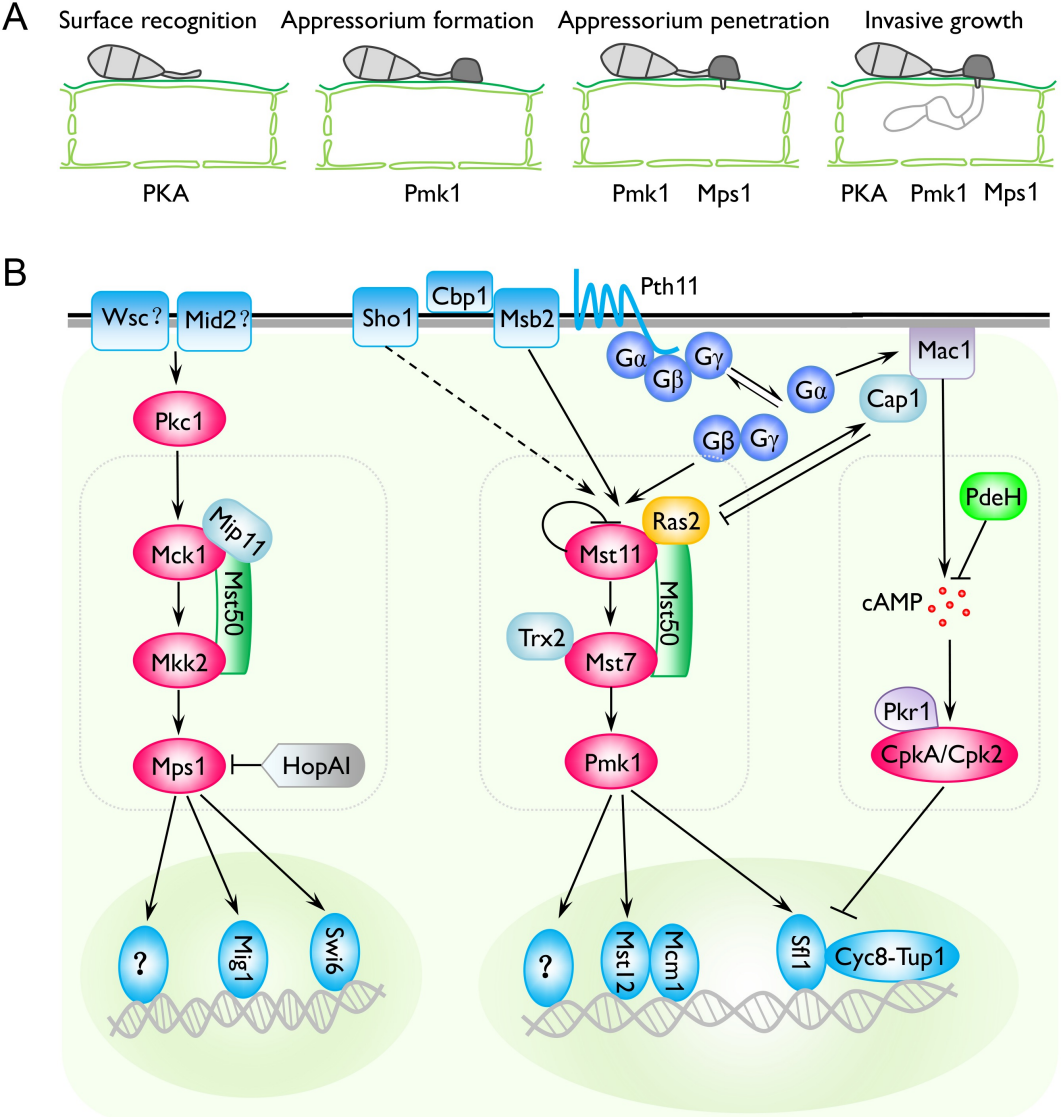
The Pmk1 and Mps1 pathways and their relationship with cAMP signaling in *Magnaporthe oryzae*. **A.** Distinct and overlapping functions of the cAMP-PKA pathway and Pmk1 and Mps1 MAPK cascades during plant infection. **B.** Physical and chemical signals known to trigger appressorium formation include surface hydrophobicity and hardness, cutin monomers, plant surface waxes, and primary alcohols. Msb2, Sho1, Pth11, and Cbp1 are involved in recognizing extracellular or surface signals to activate the downstream cAMP-PKA pathway and Mst11-Mst7-Pmk1 MAPK cascade. Both the trimeric G-proteins and Ras monomeric G-proteins are functionally related to these two pathways that regulate appressorium formation, penetration, and invasive growth. Although its upstream sensors have not been characterized, the Bck1-Mkk2-Mps1 cascade likely functions downstream from PKC and is important for sporulation, appressorium penetration, and pathogenesis via downstream Mig1, Swi6, and possibly other transcription factors. The adapter protein Mst50 is involved in both Pmk1 and Mps1 pathways. Mip11 is a RACK protein that interacts with both Mst50 and Mck1. Pmk1 positively regulates Mst12, Mcm1, Sfl1, and likely other transcription factors during different infection processes. Together with the Cyc8-Tup1 corepressor complex, Sfl1 also functions as a transcriptional repressor for hyphal growth. cAMP, cyclic adenosine monophosphate; MAPK, MAP kinase; PKA, protein kinase A; PKC, protein kinase C; RACK, receptor for activated C kinase.

Most filamentous ascomycetes have only one ortholog of yeast Fus3 and Kss1 MAPKs that function downstream from Ste11–Ste7 in the pheromone response and filamentation pathways. In over 20 plant pathogenic fungi characterized, this MAPK is important for plant infection [1,2]. In the rice blast fungus *Magnaporthe oryzae*, pathogenicity MAP kinase 1 (*PMK1*) is essential for appressorium formation and invasive growth (Fig 1A). Its ortholog is also required for appressorium formation in all the other appressorium-forming pathogens studied (Table 1). Expression of its orthologs from fungi such as *Colletotrichum lagenarium* and *Puccinia striiformis* rescues the appressorium formation defect of *pmk1* mutant, indicating the well-conserved nature of this MAPK (S1 Table). In non-appressorium-forming pathogens, this pathway is also important for plant penetration and infectious growth in various fungi, such as biotrophic *Claviceps purpurea*, hemibiotrophic *Mycosphaerella graminicola*, and necrotrophic *Stagonospora nodorum* [1,2]. *kss1* mutants of the multihost pathogen *Fusarium oxysporum* are nonpathogenic on tomato plants but fully pathogenic in a murine model system [3], indicating that this MAPK plays different roles in plant and animal pathogenesis. In *U*. *maydis*, although Kpp6 plays a more important role in appressorium penetration than Kpp2, both *kpp2* and *kpp6* mutants are attenuated in virulence, and *kpp2 kpp6* double mutants are nonpathogenic [4]. Another putative MAPK, Crk1, that was first identified as a homolog of yeast Ime2 also regulates morphogenesis and plant infection in *U*. *maydis* [5].

**Table 1:** MAP kinases characterized in plant pathogenic fungi.

Fungal species	Orthologs of yeast		
	Fus3/Kss1	Slt2	Hog1
Alternaria alternata*	Fus3	Slt2	
*A*. brassicicola*	Amk1		
*Bipolaris oryzae*	Bmk1		Srm1
Blumeria graminis*	Mpk1		
Botrytis cinerea*	Bmp1	Bmp3	Sak1
*Claviceps purpurea*	Cpmk1		
Cochliobolus heterostrophus*	Chk1		
Cochliobolus sativus*	Fus3	Slt2	Cshog1
Colletotrichum gloeosporioides*	CgMk1	Cgl-Slt2	
Colletotrichum higginsianum*	ChMK1		
Colletotrichum lagenarium*	Cmk1	Maf1	
Colletotrichum orbiculare*			Osc1
*Fusarium graminearum*	Gpmk1	Mgv1	FgHog1
*F*. *oxysporum*	Fmk1	Mpk1	
*F*. *verticillioides*	Mk1		
*Heterobasidion annosum*			HaHog1
Magnaporthe oryzae*	Pmk1	Mps1	Osm1
*Mycosphaerella graminicola*	Fus3	MgSlt2	MgHog1
*Puccinia striiformis*	Mapk1		
Pyrenophora teres*	Ptk1		
Sclerotinia sclerotiorum*	Smk1	Smk3	
Setosphaeria turcica*	Stk2		
*Stagonospora nodorum*	Mak2		
*Ustilaginoidea virens*			UvHog1
*Ustilago maydis*	Kpp2 Kpp6		UmHog1
Verticillium dahliae*	Vmk1		VdHog1

* Pathogens that form appressoria during plant infection.

**Abbreviations:** MAP, mitogen-activated protein.

Unlike its conserved role in pathogenesis, the functions of this MAPK in other development processes vary among different fungi [1,2]. For example, the Fus3 ortholog is important for deoxynivalenol production in *F*. *graminearum* and fumonisin biosynthesis in *F*. *verticillioides*, indicating a regulatory role in secondary metabolism. Whereas this MAPK is important for conidiation in *Alternaria brassicicola* and conidium germination in *C*. *lagenarium*, its ortholog regulates pycnidium formation and sclerotium development in *M*. *graminicola* and *Sclerotinia sclerotiorum* (S1 Table). In *A*. *alternata*, AsFus3 is important for conidiation and copper fungicide resistance.

In *M*. *oryzae*, Pmk1 is activated by its upstream MEK Mst7 and MEKK Mst11 (Fig 1B). Without a yeast Ste5 ortholog, Mst50 functions as an adaptor protein for the Mst11-Mst7 interaction, but Mst7 interacts with Pmk1 via its MAPK-docking site [1]. The formation of Mst7 homodimers involves the thioredoxins and is important for Pmk1 activation [6]. Besides its intramolecular self-inhibitory binding, Mst11 also interacts with Ras proteins via the Ras-association domain for Pmk1 activation [7,8]. Upstream components of this MAPK pathway also have been characterized in other fungal pathogens [1,9]. In *U*. *maydis*, the Ste50 ortholog Ubc2 functions as an adaptor protein for the Kpp4–Fuz7–Kpp2/Kpp6 cascade [10]. Orthologs of Ste12, a downstream transcription factor of yeast Fus3 and Kss1, also play a critical role in development and pathogenesis in fungal pathogens [1]. In *M*. *oryzae* and *C*. *lagenarium*, deletion of Ste12 ortholog results in the loss of pathogenicity and defects in appressorium penetration.

## The Slt2 cell wall integrity pathway also has a conserved role in pathogenesis

The MAPK cascade orthologous to the yeast Bck1–Mkk1/Mkk2–Slt2 is conserved in filamentous ascomycetes to regulate cell wall integrity (CWI) and pathogenesis [1,2]. In *M*. *oryzae*, Mps1 is essential for plant infection, and *mps1* mutant is defective in appressorium penetration. Expression of HopAI, a *Pseudomonas* MAPK–inactivating effector, strongly affects Mps1 phosphorylation and virulence [11]. The CWI MAPK pathway is also important for pathogenesis in other plant pathogens (Table 1). However, its role in appressorium formation or initial penetration varies among different fungi. Although its ortholog is important for early stages of appressorium development in *C*. *lagenarium* and *Colletotrichum gloeosporioides*, Mps1 is dispensable for appressorium formation in *M*. *oryzae*. In *M*. *graminicola*, *MgSlt2* mutant is normal in stomata penetration but defective in developing invasive hyphae.

Beside their conserved roles in infection and cell wall integrity, Slt2 orthologs also are involved in regulating different biological processes in plant pathogenic fungi. For example, the *mps1* mutant is normal in growth rate but has severe defects in aerial hyphal growth and conidiation in *M*. *oryzae*. In *S*. *sclerotiorum*, *smk3* mutant is reduced in sclerotium formation but increased in aerial hyphal growth [12]. Whereas *M*. *graminicola Mgslt2* mutant is hypersensitive to azole fungicides, *Botrytis cinerea bmp3* mutant has increased sensitivity to paraquat and fludioxonil (S1 Table).

A number of upstream and downstream components of the CWI pathway have been functionally characterized in *M*. *oryzae*, *F*. *graminearum*, and other fungi [1,9]. Whereas most plant pathogens have a single MAPK, MEK, and MEKK, a species-specific duplication of the MEKK is observed in *F*. *oxysporum* and two rust fungi. The *bck1* mutant of *Cryphonectria parasitica* has severe growth defects and often produces fast-growing sectors although the underlying mechanism is not clear [13]. For the downstream targets of the CWI MAPK cascade, the orthologs of yeast Rlm1 and Swi6 are conserved in filamentous ascomycetes. In *M*. *oryzae*, the *mig1* mutant deleted of the MCM1/AGAMOUS/DEFICIENS/SRF (MADS) box transcription factor orthologous to Rlm1 is normal in vegetative growth and the formation of melanized appressoria but defective in the differentiation and growth of invasive hyphae. The *Moswi6* mutant deleted of the transcriptional regulator orthologous to yeast Swi6 is defective in cell wall integrity, hyphal growth, and appressorium penetration [1].

## The osmoregulation pathway plays a species-specific role in pathogenesis

**Fig 2:**
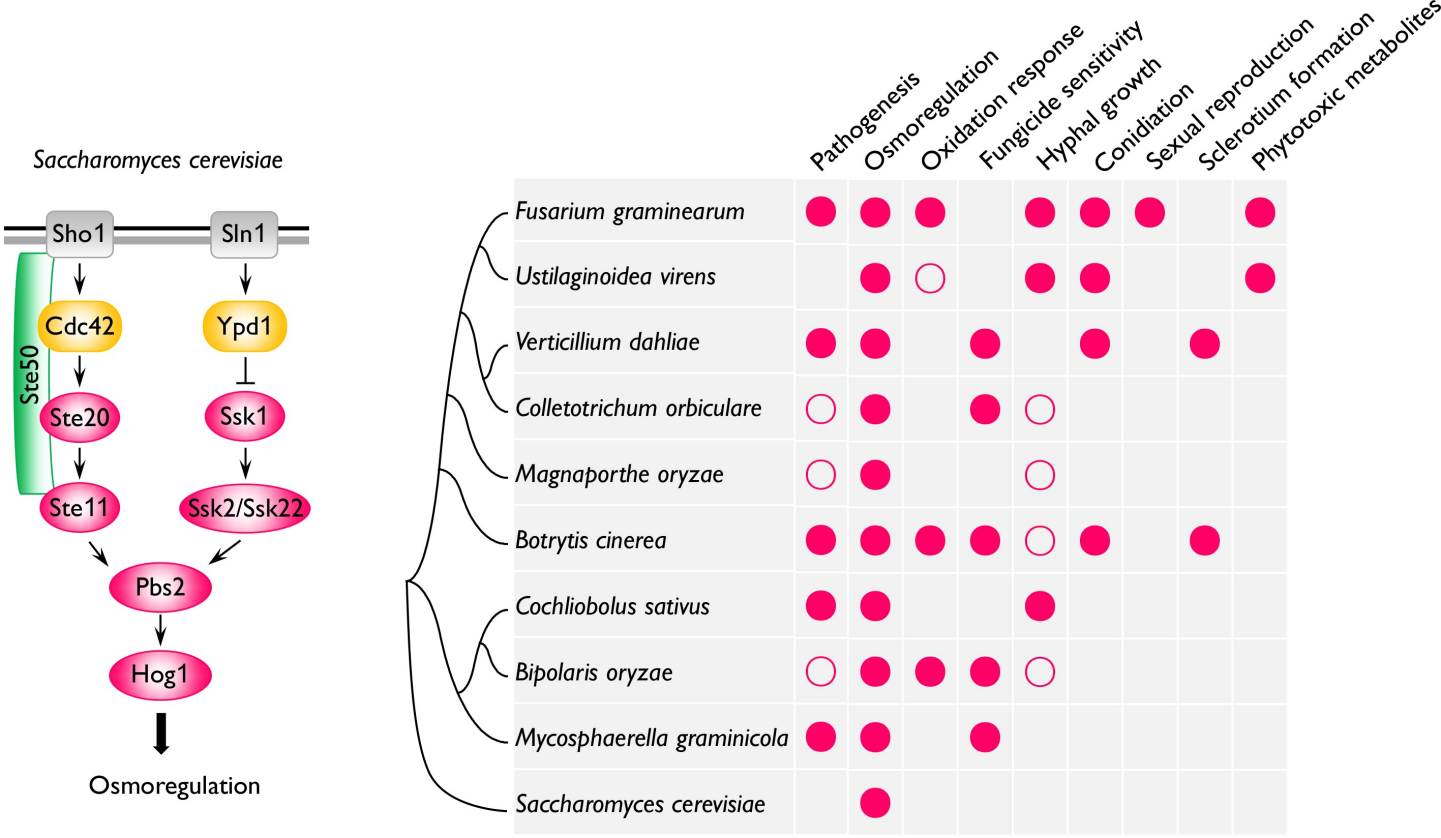
Functional diversity of the HOG pathway in yeast and plant-pathogenic fungi. Schematic model of the HOG pathway that is mainly involved in hyperosmoregulation in *S*. *cerevisiae*. Although lacking redundant MEK kinases, filamentous ascomycetes generally have orthologs of all these conserved components of the Hog1 pathway, including MEKK, MEK, and upstream phosphorelay and sensor proteins. Besides its conserved role in osmoregulation, this pathway has species-specific functions in pathogenesis, vegetative growth, fungicide sensitivity, sexual and asexual development, and responses to oxidative, cell wall, and other stresses in different plant pathogenic fungi. Filled and empty circles indicate that the Hog1/OS-2 kinase is important or dispensable, respectively, for specific functions characterized in different fungi. HOG, high-osmolarity glycerol; MEK, MAPK kinase; MEKK, MEK kinase.

Unlike the other two MAPKs with the threonine-glutamate-tyrosine (TEY) phosphorylation sites, the Hog1 or OS-2 ortholog with the threonine-glycine-tyrosine (TGY) phosphorylation sites is not important for pathogenesis in all the plant pathogenic fungi (Fig 2). In *M*. *oryzae*, Osm1 is dispensable for appressorium turgor generation and pathogenesis. Hog1 ortholog is also dispensable for plant infection in *Cochliobolus orbiculare* and *Bipolaris oryzae* [1,2]. However, mutants blocked in this pathway are defective in plant infection in *B*. *cinerea*, *M*. *graminicola*, and other fungi (S1 Table). Whereas *Mghog1* mutant is nonpathogenic and defective in the yeast-like-to-hyphal growth switch in *M*. *graminicola*, deletion of this MAPK reduces the production of phytotoxic metabolites in *F*. *graminearum* and *Ustilaginoidea virens* [1,14]. The *Cochliobolus sativus Cshog1* mutant is normal in root infection but significantly reduced in virulence on barley leaves [15]. Therefore, the function of this MAPK pathway in pathogenesis may be not only species-specific but also tissue-specific.

In phytopathogenic fungi, the Hog1 pathway also plays a species-specific role in growth and development, such as the regulation of perithecium formation in *F*. *graminearum* and microsclerotium formation in *Verticillium dahliae* (S1 Table). In *B*. *cinerea*, Sak1 positively controls conidiation but negatively regulates sclerotium development. Nevertheless, in general, this TGY MAPK is important for oxidative stress and responsible for sensitivity to phenylpyrrole fungicides, although its role in response to cell wall and other stresses may vary among different fungi. The upstream MEK and MEKK and downstream transcription factors of the Hog1 pathway also have been characterized in several fungal pathogens [1,2,16]. Deletion of FgPbs2 and FgSsk22 results in the same defects as *Fgos2* mutant in *F*. *graminearum* [9], in which the ATF/CREB transcription factor FgAtf1 interacts with FgOs2 in the nucleus under osmotic stress and constitutive expression of *FgATF1* almost fully complements *Fgos2* defects in osmoregulation and pathogenesis [17].

## Upstream receptors or sensors

Unlike plants and animals, fungi lack receptor kinases or receptor-like kinases. However, in comparison with yeast, G-protein–coupled receptor (GPCR) genes are expanded in plant pathogens, such as 76 and 116 putative GPCR genes in *M*. *oryzae* and *F*. *graminearum*, respectively. In *M*. *oryzae*, Pth11, a noncanonical GPCR with the CFEM motif, is involved in surface recognition for appressorium formation [18]. It is internalized and transported to dynamic tubulovesicular endosomal compartments with its downstream signaling components [1,19]. In plant pathogens, CFEM domain–containing GPCRs are often induced during plant infection [20]. In *F*. *oxysporum*, the ortholog of yeast Ste2 pheromone receptor appears to be involved in the sensing of α-pheromone, peroxidase, and other host compounds [21]. However, deletion of Ste2 ortholog has no obvious effect on virulence.

The other two receptor-like genes functioning upstream from MAPK pathways in plant pathogens are orthologous to yeast Msb2 and Sho1 [1,2]. In *U*. *maydis* and *F*. *oxysporum*, Msb2 plays a major and Sho1 plays a minor role in activating downstream MAPKs and pathogenesis. In *M*. *oryzae*, the *Momsb2 Mosho1* mutant rarely forms appressoria on artificial hydrophobic surfaces but still develops appressoria in response to plant surface waxes and primary alcohols. MoMsb2 is functionally related to another mucin-like protein, MoCbp1, because *Momsb2 Mocbp1* mutant is defective in Pmk1 activation and non-pathogenic [22]. The Msb2 ortholog is also important for plant infection in *V*. *dahliae* but not in *B*. *cinerea*, although Bmp1 MAPK is activated in a Msb2-dependent manner [23].

No receptors have been characterized for the CWI pathway in plant pathogenic fungi, although they have Wsc1 and Mid1 homologs. In contrast, components of the two-component phosphorelay system upstream from the Hog1 cascade have been characterized in several fungal pathogens [1,2]. In *M*. *oryzae*, the two histidine kinases—MoSln1 and MoHik1—differ in sensing salt and sugar stresses, but both of them are important for full virulence. MoYpd1, the only intermediate signal transfer protein, is important for pathogenesis and hyperactivation of Osm1 in response to fungicides and osmotic stress.

## Cross-talking among different MAPK pathways

In *Cochliobolus heterostrophus*, Chk1 and Mps1 coregulate several downstream targets, such as the *Colletotrichum* melanin regulation (*CMR1*) transcription factor and melanin biosynthesis genes. Hog1 plays an opposite role in the regulation of some Chk1 targets, although it has overlapping functions with Chk1 during plant infection [24]. In *F*. *oxysporum*, both Fmk1 and Mpk1 regulate responses to cell wall and heat stresses, and Hog1 likely negatively controls the activation of Fmk1 and Mpk1 [25]. In *M*. *oryzae*, besides its role in the Pmk1 pathway, Mst50 interacts with Mck1 and Mkk2, and the *mst50* mutant is defective in Mps1 phosphorylation under cell wall stress. Deletion of *MST50* also affects Osm1 activation in response to hyperosmotic stress, and Hik1 interacts with Mst50 [26].

Because the cyclic adenosine monophosphate–protein kinase A (cAMP-PKA) pathway also regulates various developmental and infection processes, cross-talking between cyclic adenosine monophosphate (cAMP) signaling and MAPK cascades must occur and likely involve different mechanisms in plant pathogenic fungi [1,2,27]. In *S*. *cerevisiae*, inhibition of β-1,3-glucan synthesis led to the activation of the CWI pathway and suppression of protein kinase A (PKA) signaling [28]. In *U*. *maydis*, the Prf1 transcription factor functions downstream from both the cAMP-PKA and MAPK pathways. In *M*. *oryzae*, MoRas2 functions upstream from both cAMP signaling and Pmk1 cascade (Fig 1B). Loss-of-function mutations in MoSfl1, a Pmk1-interacting transcription factor, suppress the growth defects of *cpkA cpk2* mutant [29].

Overall, plant pathogenic fungi must properly respond to host and environmental signals throughout the infection cycle. Further characterization of cross-talking among MAPK cascades or their relationships with other signaling pathways will lead to better understanding of the regulatory network involved in the regulation of different infection processes. Another important area is to identify and characterize the upstream receptors by systematic characterization of GPCRs. The expansion of GPCR genes in plant pathogenic fungi strongly suggests their importance for the recognition of extracellular cues, although many of them may have overlapping functions. Furthermore, MAPK pathways may regulate other biological processes that have not been well studied in plant pathogens, such as response to volatile signals.

## Supporting information

S1 Table Three MAP kinases characterized in different plant pathogens. (DOCX)
