# Supplementary material for: Mitogen-activated protein kinase signaling in plant pathogenic fungi
Source: PLoS Pathog. 2018 Mar 15;14(3):e1006875. doi: 10.1371/journal.ppat.1006875 (PMC5854419; doi:10.1371/journal.ppat.1006875)
Supplement: S1 Table — (DOCX) [file ppat.1006875.s001.docx]

**Supplemental Table 1. Three MAP kinases characterized in different plant pathogens**

| **Fungal Species** | **References** |
| --- | --- |
| **Yeast Fus3/Kss1 ortholog** | |
| *Alternaria alternata* | [[1](#_ENREF_1)] |
| *Alternaria brassicicola* | [[2](#_ENREF_2)] |
| *Bipolaris oryzae* | [[3](#_ENREF_3)] |
| *Blumeria graminis* | [[4](#_ENREF_4)] |
| *Botrytis cinerea* | [[5](#_ENREF_5),[6](#_ENREF_6)] |
| *Cochliobolus heterostrophus* | [[7](#_ENREF_7)] |
| [*Cochliobolus sativus*](https://en.wikipedia.org/wiki/Cochliobolus_sativus) | [[8](#_ENREF_8)] |
| *Colletotrichum gloeosporioides* | [[9](#_ENREF_9)] |
| *Colletotrichum higginsianum* | [[10](#_ENREF_10)] |
| *Colletotrichum lagenarium* | [[11](#_ENREF_11)] |
| *Claviceps purpurea* | [[12](#_ENREF_12)] |
| *Fusarium graminearum* | [[13](#_ENREF_13)] |
| *Fusarium oxysporum* | [[14](#_ENREF_14)] |
| *Fusarium verticillioides* | [[15](#_ENREF_15)] |
| *Magnaporthe oryzae* | [[16](#_ENREF_16)] |
| *Mycosphaerella graminicola* | [[17](#_ENREF_17)] |
| *Puccinia striiformis* | [[18](#_ENREF_18)] |
| *Pyrenophora teres* | [[19](#_ENREF_19)] |
| *Sclerotinia sclerotiorum* | [[20](#_ENREF_20)] |
| *Setosphaeria turcica* | [[21](#_ENREF_21)] |
| *Stagonospora nodorum* | [[22](#_ENREF_22)] |
| *Ustilago maydis* | [[23](#_ENREF_23),[24](#_ENREF_24)] |
| *Verticillium dahliae* | [[25](#_ENREF_25)] |
| **Yeast Slt2 ortholog** | |
| *Alternaria alternata* | [[26](#_ENREF_26)] |
| *Botrytis cinerea* | [[27](#_ENREF_27)] |
| [*Cochliobolus sativus*](https://en.wikipedia.org/wiki/Cochliobolus_sativus) | [[8](#_ENREF_8)] |
| *Colletotrichum gloeosporioides* | [[28](#_ENREF_28)] |
| *Colletotrichum lagenarium* | [[29](#_ENREF_29)] |
| *Fusarium graminearum* | [[30](#_ENREF_30)] |
| *Fusarium oxysporum* | [[31](#_ENREF_31)] |
| *Magnaporthe oryzae* | [[32](#_ENREF_32)] |
| *Mycosphaerella graminicola* | [[33](#_ENREF_33)] |
| *Sclerotinia sclerotiorum* | [[34](#_ENREF_34)] |
| **Yeast Hog1 ortholog** | |
| *Bipolaris oryzae* | [[35](#_ENREF_35)] |
| *Botrytis cinerea* | [[36](#_ENREF_36),[37](#_ENREF_37)] |
| [*Cochliobolus sativus*](https://en.wikipedia.org/wiki/Cochliobolus_sativus) | [[8](#_ENREF_8)] |
| *Colletotrichum orbiculare* | [[38](#_ENREF_38)] |
| *Fusarium graminearum* | [[39](#_ENREF_39),[40](#_ENREF_40)] |
| *Heterobasidion annosum* | [[41](#_ENREF_41)] |
| *Magnaporthe oryzae* | [[42](#_ENREF_42)] |
| *Mycosphaerella graminicola* | [[43](#_ENREF_43)] |
| [*Ustilaginoidea virens*](http://cn.bing.com/academic/profile?id=39fae933f8903f242013f495ce473064&encoded=0&v=paper_preview&mkt=zh-cn) | [[44](#_ENREF_44)] |
| *Verticillium dahliae* | [[45](#_ENREF_45)] |

**REFERENCES**

1. Lin CH, Yang SL, Wang NY, Chung KR (2010) The *FUS3* MAPK signaling pathway of the citrus pathogen *Alternaria alternata* functions independently or cooperatively with the fungal redox-responsive *AP1* regulator for diverse developmental, physiological and pathogenic processes. Fungal Genet Biol 47: 381-391.

2. Cho Y, Cramer RA, Kim KH, Davis J, Mitchell TK, et al. (2007) The Fus3/Kss1 MAP kinase homolog Amk1 regulates the expression of genes encoding hydrolytic enzymes in *Alternaria brassicicola*. Fungal Genet Biol 44: 543-553.

3. Moriwaki A, Kihara J, Mori C, Arase S (2007) A MAP kinase gene, *BMK1*, is required for conidiation and pathogenicity in the rice leaf spot pathogen *Bipolaris oryzae*. Microbiol Res 162: 108-114.

4. Zhang ZG, Gurr SJ (2001) Expression and sequence analysis of the *Blumeria graminis* mitogen-activated protein kinase genes, *mpk1* and *mpk2*. Gene 266: 57-65.

5. Zheng L, Campbell M, Murphy J, Lam S, Xu JR (2000) The *BMP1* gene is essential for pathogenicity in the gray mold fungus *Botrytis cinerea*. Mol Plant Microbe In 13: 724-732.

6. Leroch M, Mueller N, Hinsenkamp I, Hahn M (2015) The signalling mucin Msb2 regulates surface sensing and host penetration via *BMP1* MAP kinase signalling in *Botrytis cinerea*. Mol Plant Pathol 16: 787-798.

7. Lev S, Sharon A, Hadar R, Ma H, Horwitz BA (1999) A mitogen-activated protein kinase of the corn leaf pathogen *Cochliobolus heterostrophus* is involved in conidiation, appressorium formation, and pathogenicity: Diverse roles for mitogen-activated protein kinase homologs in foliar pathogens. Proc Natl Acad Sci U S A 96: 13542-13547.

8. Leng YQ, Zhong SB (2015) The role of mitogen-activated protein (MAP) kinase signaling components in the fungal development, stress response and virulence of the fungal cereal pathogen *Bipolaris sorokiniana*. PLoS One 10: e0128291.

9. He P, Wang Y, Wang X, Zhang X, Tian C (2017) The mitogen-activated protein kinase *CgMK1* governs appressorium formation, melanin synthesis, and plant infection of *Colletotrichum gloeosporioides*. Front Microbiol 8: 2216.

10. Wei W, Xiong Y, Zhu W, Wang N, Yang G, et al. (2016) *Colletotrichum higginsianum* mitogen-activated protein kinase *ChMK1*: role in growth, cell Wall Integrity, colony melanization, and pathogenicity. Front Microbiol 7: 1212.

11. Takano Y, Kikuchi T, Kubo Y, Hamer JE, Mise K, et al. (2000) The *Colletotrichum lagenarium* MAP kinase gene *CMK1* regulates diverse aspects of fungal pathogenesis. Mol Plant Microbe In 13: 374-383.

12. Mey G, Oeser B, Lebrun MH, Tudzynski P (2002) The biotrophic, non-appressorium-forming grass pathogen *Claviceps purpurea* needs a Fus3/Pmk1 homologous mitogen-activated protein kinase for colonization of rye ovarian tissue. Mol Plant Microbe In 15: 303-312.

13. Jenczmionka NJ, Maier FJ, Losch AP, Schafer W (2003) Mating, conidiation and pathogenicity of *Fusarium graminearum*, the main causal agent of the head-blight disease of wheat, are regulated by the MAP kinase *gpmk1*. Curr Genet 43: 87-95.

14. Di Pietro A, Garcia-Maceira FI, Meglecz E, Roncero MIG (2001) A MAP kinase of the vascular wilt fungus *Fusarium oxysporum* is essential for root penetration and pathogenesis. Mol Microbiol 39: 1140-1152.

15. Zhang Y, Choi YE, Zou X, Xu JR (2011) The *FvMK1* mitogen-activated protein kinase gene regulates conidiation, pathogenesis, and fumonisin production in *Fusarium verticillioides*. Fungal Genet Biol 48: 71-79.

16. Xu JR, Hamer JE (1996) MAP kinase and cAMP signaling regulate infection structure formation and pathogenic growth in the rice blast fungus *Magnaporthe grisea*. Genes Dev 10: 2696-2706.

17. Cousin A, Mehrabi R, Guilleroux M, Dufresne M, Van der Lee T, et al. (2006) The MAP kinase-encoding gene MgFus3 of the non-appressorium phytopathogen *Mycosphaerella graminicola* is required for penetration and in vitro pycnidia formation. Mol Plant Pathol 7: 269-278.

18. Guo J, Dai XW, Xu JR, Wang YL, Bai PF, et al. (2011) Molecular Characterization of a Fus3/Kss1 Type MAPK from *Puccinia striiformis* f. sp *tritici*, *PsMAPK1*. PLoS One 6: e21895.

19. Ruiz-Roldan MC, Maier FJ, Schafer W (2001) *PTK1*, a mitogen-activated-protein kinase gene, is required for conidiation, appressorium formation, and pathogenicity of *Pyrenophora teres* on barley. Mol Plant Microbe In 14: 116-125.

20. Chen CB, Harel A, Gorovoits R, Yarden O, Dickman MB (2004) MAPK regulation of sclerotial development in *Sclerotinia sclerotiorum* is linked with pH and cAMP sensing. Mol Plant Microbe In 17: 404-413.

21. Gu SQ, Yang Y, Li P, Zhang CZ, Fan Y, et al. (2013) Stk2, a mitogen-activated protein kinase from *Setosphaeria turcica*, specifically complements the functions of the Fus3 and Kss1 of *Saccharomyces cerevisiae* in filamentation, invasive growth, and mating behavior. J Integr Agr 12: 2209-2216.

22. Solomon PS, Waters ODC, Simmonds J, Cooper RM, Oliver RP (2005) The Mak2 MAP kinase signal transduction pathway is required for pathogenicity in *Stagonospora nodorum*. Curr Genet 48: 60-68.

23. Muller P, Aichinger C, Feldbrugge M, Kahmann R (1999) The MAP kinase Kpp2 regulates mating and pathogenic development in *Ustilago maydis*. Mol Microbiol 34: 1007-1017.

24. Brachmann A, Schirawski J, Muller P, Kahmann R (2003) An unusual MAP kinase is required for efficient penetration of the plant surface by *Ustilago maydis*. EMBO J 22: 2199-2210.

25. Rauyaree P, Ospina-Giraldo MD, Kang S, Bhat RG, Subbarao KV, et al. (2005) Mutations in *VMK1*, a mitogen-activated protein kinase gene, affect microsclerotia formation and pathogenicity in *Verticillium dahliae*. Curr Genet 48: 109-116.

26. Yago JI, Lin CH, Chung KR (2011) The SLT2 mitogen-activated protein kinase-mediated signalling pathway governs conidiation, morphogenesis, fungal virulence and production of toxin and melanin in the tangerine pathotype of *Alternaria alternata*. Mol Plant Pathol 12: 653-665.

27. Rui O, Hahn M (2007) The Slt2-type MAP kinase Bmp3 of *Botrytis cinerea* is required for normal saprotrophic growth, conidiation, plant surface sensing and host tissue colonization. Mol Plant Pathol 8: 173-184.

28. Yong HY, Bakar FDA, Illias RM, Mahadi NM, Murad AMA (2013) *Cgl-SLT2* is required for appressorium formation, sporulation and pathogenicity in *Colletotrichum gloeosporioides*. Braz J Microbiol 44: 1241-1250.

29. Kojima K, Kikuchi T, Takano Y, Oshiro E, Okuno T (2002) The mitogen-activated protein kinase gene MAF1 is essential for the early differentiation phase of appressorium formation in *Colletotrichum lagenarium*. Mol Plant Microbe In 15: 1268-1276.

30. Hou Z, Xue C, Peng Y, Katan T, Kistler HC, et al. (2002) A mitogen-activated protein kinase gene (MGV1) in *Fusarium graminearum* is required for female fertility, heterokaryon formation, and plant infection. Mol Plant Microbe In 15: 1119-1127.

31. Segorbe D, Di Pietro A, Perez-Nadales E, Turra D (2017) Three *Fusarium oxysporum* mitogen-activated protein kinases (MAPKs) have distinct and complementary roles in stress adaptation and cross-kingdom pathogenicity. Mol Plant Pathol 18: 912-924.

32. Xu JR, Staiger CJ, Hamer JE (1998) Inactivation of the mitogen-activated protein kinase Mps1 from the rice blast fungus prevents penetration of host cells but allows activation of plant defense responses. Proc Natl Acad Sci U S A 95: 12713-12718.

33. Mehrabi R, van der Lee T, Waalwijk C, Kema GHJ (2006) MgSlt2, a cellular integrity MAP kinase gene of the fungal wheat pathogen *Mycosphaerella graminicola*, is dispensable for penetration but essential for invasive growth. Mol Plant Microbe In 19: 389-398.

34. Bashi ZD, Gyawali S, Bekkaoui D, Coutu C, Lee L, et al. (2016) The *Sclerotinia sclerotiorum* Slt2 mitogen-activated protein kinase ortholog, *SMK3*, is required for infection initiation but not lesion expansion. Can J Microbiol 62: 836-850.

35. Moriwaki A, Kubo E, Arase S, Kihara J (2006) Disruption of *SRM1*, a mitogen-activated protein kinase gene, affects sensitivity to osmotic and ultraviolet stressors in the phytopathogenic fungus *Bipolaris oryzae*. Fems Microbiol Lett 257: 253-261.

36. Heller J, Ruhnke N, Espino JJ, Massaroli M, Collado IG, et al. (2012) The mitogen-activated protein kinase BcSak1 of *Botrytis cinerea* is required for pathogenic development and has broad regulatory functions beyond stress response. Mol Plant Microbe In 25: 802-816.

37. Liu W, Leroux P, Fillinger S (2008) The *HOG1*-like MAP kinase Sak1 of *Botrytis cinerea* is negatively regulated by the upstream histidine kinase Bos1 and is not involved in dicarboximide- and phenylpyrrole-resistance. Fungal Genet Biol 45: 1062-1074.

38. Kojima K, Takano Y, Yoshimi A, Tanaka C, Kikuchi T, et al. (2004) Fungicide activity through activation of a fungal signalling pathway. Mol Microbiol 53: 1785-1796.

39. Zheng DW, Zhang SJ, Zhou XY, Wang CF, Xiang P, et al. (2012) The *FgHOG1* pathway regulates hyphal growth, stress responses, and plant infection in *Fusarium graminearum*. PLoS One 7: e49495.

40. Nguyen TV, Schafer W, Bormann J (2012) The stress-activated protein kinase *FgOS-2* is a key regulator in the life cycle of the cereal pathogen *Fusarium graminearum*. Mol Plant Microbe In 25: 1142-1156.

41. Raffaello T, Kerio S, Asiegbu FO (2012) Role of the *HaHOG1* MAP kinase in response of the conifer root and butt rot pathogen (*Heterobasidion annosum*) to osmotic and oxidative stress. PLoS One 7: e31186.

42. Dixon KP, Xu JR, Smirnoff N, Talbot NJ (1999) Independent signaling pathways regulate cellular turgor during hyperosmotic stress and appressorium-mediated plant infection by *Magnaporthe grisea*. Plant Cell 11: 2045-2058.

43. Mehrabi R, Zwiers LH, de Waard MA, Kema GH (2006) MgHog1 regulates dimorphism and pathogenicity in the fungal wheat pathogen *Mycosphaerella graminicola*. Mol Plant Microbe In 19: 1262-1269.

44. Zheng DW, Wang Y, Han Y, Xu JR, Wang CF (2016) *UvHOG1* is important for hyphal growth and stress responses in the rice false smut fungus *Ustilaginoidea virens*. Sci Rep 6: 24824.

45. Wang YL, Tian LY, Xiong DG, Klosterman SJ, Xiao SX, et al. (2016) The mitogen-activated protein kinase gene, VdHog1, regulates osmotic stress response, microsclerotia formation and virulence in *Verticillium dahliae*. Fungal Genet Biol 88: 13-23.
